# Reducing inappropriate antibiotic use in febrile neutropenia in hematology patients through the implementation of an antibiotic de-escalation protocol

**DOI:** 10.1017/ice.2025.90

**Published:** 2025-07

**Authors:** Jinghao Nicholas Ngiam, Victor Ling, Matthew Chung Yi Koh, Mohamed Nasar Fathima Rofina Farveen, Shi Hui Clarice Choong, Li Mei Michelle Poon, Liang Piu Koh, Nares Smitasin, Lionel Hon-Wai Lum

**Affiliations:** 1 Division of Infectious Diseases, Department of Medicine, National University Hospital, National University Health System, Singapore, Singapore; 2 Department of Hematology and Oncology, National University Health System, Singapore, Singapore; 3 Department of Pharmacy, National University Hospital, Singapore, Singapore; 4 Yong Loo Lin School of Medicine, National University of Singapore, Singapore, Singapore

## Abstract

**Background:**

Broad-spectrum antibiotic use in febrile neutropenia is often driven by concerns for severe and drug-resistant infections. In select patients who do not have an active infection and improve, their prolonged and unnecessary use contributes to antimicrobial resistance, drug toxicity, and increased healthcare costs. We describe the implementation of an antibiotic de-escalation protocol to reduce inappropriate antibiotic use in febrile neutropenia among hematology patients.

**Methods:**

We conducted baseline analysis (January–June 2024) of antibiotic use in febrile neutropenia cases admitted under hematology. Interventions included the (i) development of an antibiotic de-escalation protocol to guide clinical management, (ii) a roadshow to educate and improve uptake of this protocol, and (iii) regular feedback via “report cards” for hematology teams. The primary outcome was the proportion of febrile neutropenia cases with inappropriate antibiotic use, with secondary measures including adverse outcomes (in-hospital mortality, *Clostridioides difficile* infection, need for intensive care).

**Results:**

Baseline data indicated inappropriate antibiotic use rates of 45.5–66.7% per month from January to June 2024, with 13–28 days of inappropriate therapy. The protocol was developed in July 2024, with a subsequent roadshow to promote its uptake. Regular feedback was provided in the form of “report cards” every 2-monthly thereafter. Post-intervention, inappropriate antibiotic use decreased to a median of 23.35% from July to December 2024, with no observed increase in adverse outcomes.

**Conclusions:**

The implementation of a structured de-escalation protocol, combined with frequent education and feedback, effectively reduced inappropriate antibiotic use in febrile neutropenia without compromising patient safety.

## Introduction

Febrile neutropenia is a common and potentially life-threatening complication in hematology patients, which may be contributed to by the underlying disease state, and chemotherapy. Because of compromised immunity, the management of this condition often necessitates the prompt initiation of broad-spectrum antibiotics to mitigate the risk of adverse outcomes from severe infections.^1^ However, the prolonged and indiscriminate use of these agents can lead to significant adverse consequences, including the emergence of antimicrobial resistance, increased drug-related toxicities, and elevated healthcare costs.^2^ Therefore, antimicrobial stewardship programs have an important role to play in emphasizing judicious antibiotic use without compromising patient safety.^3^

Hematology patients represent a particularly vulnerable population due to their profound immunosuppression and prolonged periods of neutropenia. These patients often experience high consumption of broad-spectrum antimicrobial therapy, which in part leads to the high prevalence of multidrug-resistant organisms (MDROs) in this group.^4,5^ To limit the exposure to broad-spectrum antibiotics, experts and guidelines recommend early and prompt antimicrobial de-escalation strategies in clinically stable patients.^6^ Furthermore, there are also several noninfectious causes of fever amongst patients with febrile neutropenia, in whom broad-spectrum antibiotics are not indicated.^7^ However, real-world implementation remains limited. Possible barriers to the uptake of this strategy include fear of adverse outcomes, variability in clinician familiarity with protocols, and systemic issues such as time constraints and lack of access to guidelines during clinical decision-making.^8^

This quality improvement project was initiated at our single tertiary institution as a collaboration among infectious diseases physicians, pharmacists, and hematologists to address these challenges. The objectives were to reduce inappropriate antibiotic use among febrile neutropenia patients admitted under hematology through the implementation of a structured antibiotic de-escalation protocol. The protocol was supported by targeted educational interventions and a feedback mechanism to promote adherence. This manuscript details the development, implementation, and outcomes of the project, highlighting its role in advancing antimicrobial stewardship and optimizing patient care in this high-risk population.

## Methods

### Setting and baseline analysis

The project was conducted in a single tertiary institution, involving patients admitted to hematology with a diagnosis of febrile neutropenia from January to December 2024. Baseline data (January–June 2024) was reviewed to assess antibiotic use in febrile neutropenia cases. We screened for these cases on a daily basis. Inclusion criteria included adult hematology patients admitted with febrile neutropenia, defined as fever (temperature >38 °C) during neutropenia (absolute neutrophil count <0.5 × 10^9^/L). The index episode of febrile neutropenia for each hospital admission was considered for evaluation. Cases were evaluated by infectious diseases physicians for the appropriateness of antibiotic use (MCYK, JNN, LHWL). Initial empiric broad-spectrum antibiotics were appropriate (usually piperacillin-tazobactam, cefepime or meropenem), but should be de-escalated based on clinical response/stability, or adjusted in response to the microbiological findings. Inappropriate antibiotic use was defined in four categories, namely, (i) if an alternate diagnosis was found, and antibiotics were no longer required; (ii) if there was clinical improvement with defervescence at 96 hours and no positive microbiological studies; (iii) if the antibiotic choice had been too broad, and not tailored according to antimicrobial susceptibility testing results; and (iv) if the antibiotic duration was too prolonged for the clinical syndrome. Appropriate antibiotic duration was defined in a separate document that was also available to physicians within the hospital intranet (for example, it specifies the appropriate antibiotic duration as 5–7 days for pneumonia, and 5 days for an uncomplicated skin and soft tissue infection).

Focus groups were held with stakeholders, including the junior doctors and attending physicians in the hematology department, to identify barriers to optimal antibiotic use.

### Intervention/design

**Figure 1.**
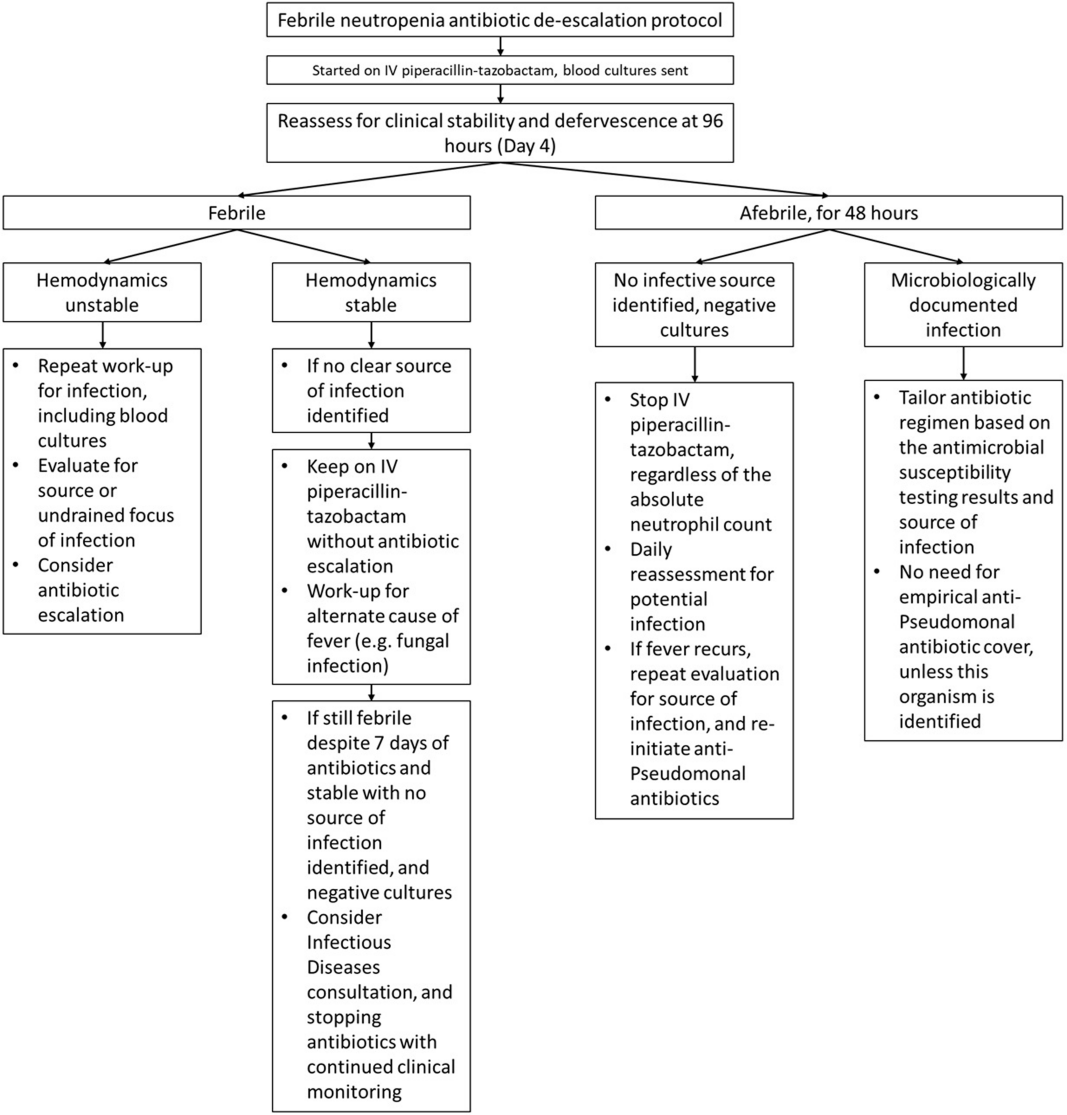
Clinical protocol for antibiotic de-escalation in febrile neutropenia.

Subsequently, interventions were developed and implemented through “Plan-Do-Study-Act” (PDSA) cycles:

Protocol Development (July 2024): A standardized antibiotic de-escalation protocol (Figure 1) was created, outlining criteria for antibiotic de-escalation, which depended on clinical improvement and microbiological findings., The protocol was developed in discussion with hematologists, infectious diseases physicians and pharmacists.

Educational Roadshow (July 2024): An educational roadshow was conducted in two separate 30-minute sessions. These sessions explained the importance and implementation of the protocol in a case-based approach. All providers who managed patients in hematology (from junior doctors, pharmacists to attending physicians) were required to attend. The slide deck and teaching materials were made available to all stakeholders after the sessions as well.

Feedback Mechanism (August 2024–December 2024): Monthly audits were conducted, and individualized “report cards” were provided to hematology teams, highlighting adherence to the protocol and outcomes.

As part of the PDSA process, we tracked the performance of each intervention, obtaining feedback from stakeholders as well at each stage, in order to refine each intervention.

### Measured outcomes and statistical analyses

The primary outcome was the proportion of febrile neutropenia cases with inappropriate antibiotic use in those eligible for cessation or de-escalation of antimicrobials based on the newly defined protocol (Figure 1), amongst all the cases of febrile neutropenia admitted to our institution in this time period.

We recorded the clinical outcomes for each patient on a monthly basis, through access of the electronic medical records, and presented the outcomes as frequencies and percentages. We tabulated the secondary outcomes including the reasons for inappropriate antibiotic use, and adverse outcomes such as in-hospital mortality, need for intensive care and *Clostridium difficile* infection. Each of these parameters were compared before and after the implementation of the intervention. We used t-tests to compare continuous parameters, while Chi-squared tests (or Fisher’s Exact test where appropriate) were used to compare categorical parameters. All statistical analyses were carried out on SPSS version 20.0 (SPSS, Inc., Chicago, Illinois). A p-value less than 0.05 was considered significant. This project was conducted as part of a quality improvement initiative and did not require individual patient/participant consent. Ethical approval for this study was obtained from the National Healthcare Group Domain Specific Review Board (NHG DSRB Ref: 2024-3902) for the access of the electronic medical records.

## Results

### Baseline findings

Between January and June 2024, 9–11 febrile neutropenia cases were identified per month. Inappropriate antibiotic use was observed in 45.5%–66.7% (median 52.25%, interquartile range, IQR 50% – 54.5%) of cases. From the qualitative data obtained from focus groups, we identified several key barriers to antibiotic de-escalation, including (i) perceived risks of recurrent fever and infection, (ii) limited familiarity with de-escalation criteria among junior staff, and (iii) systemic challenges, such as limited access to guidelines and time constraints during busy ward rounds.

### Intervention

In response, we designed a simple-to-implement antibiotic de-escalation protocol to provide clear clinical guidance on appropriate antibiotic use in patients with febrile neutropenia (Figure 1). We subsequently conducted educational roadshows (two separate 30-minute sessions, involving all junior doctors, pharmacists and attending physicians involved in the care of hematology patients) to publicize the availability of this clinical protocol. Thereafter, continual feedback was provided through monthly audits and “report cards” presented to the hematology department, indicating the percentage adherence to the protocol, and highlighting cases of appropriate and inappropriate antibiotic use.

### Post-intervention outcomes

**Figure 2.**
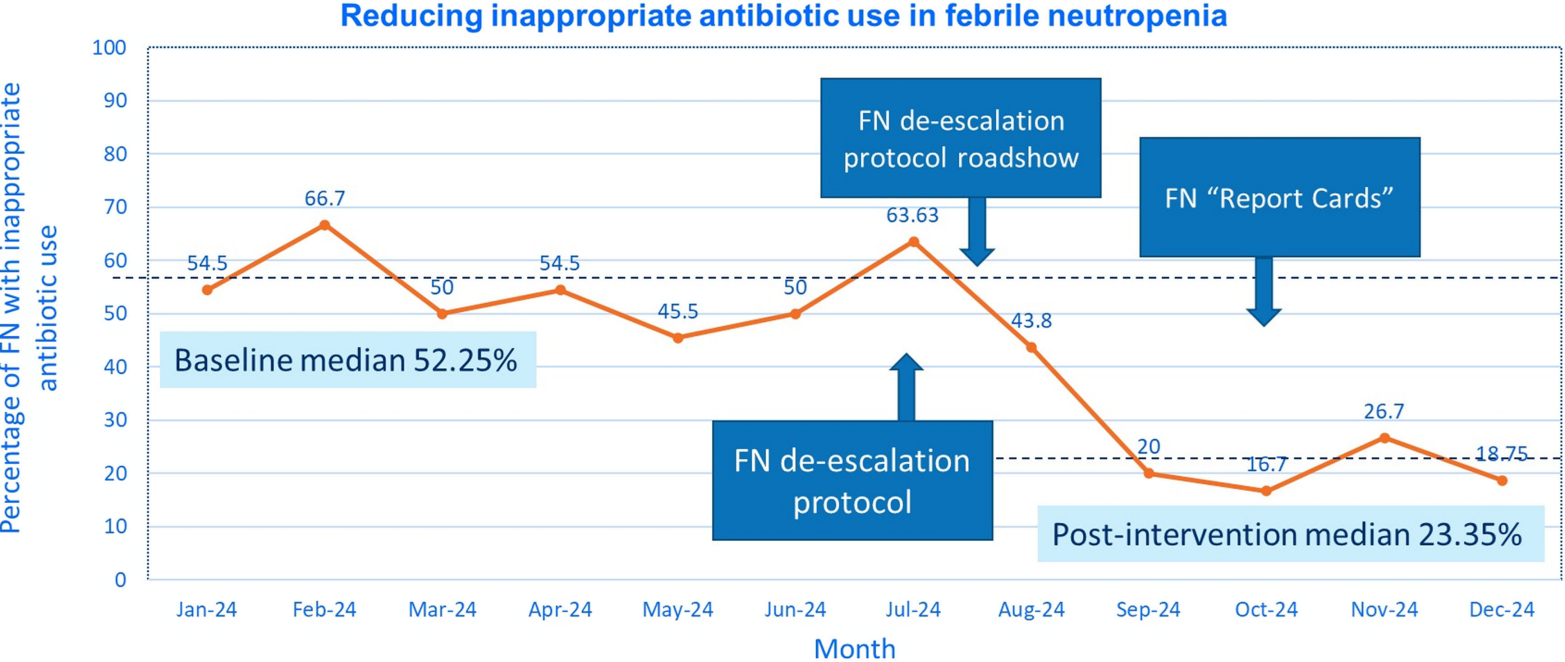
Run chart showing percentage of inappropriate antibiotic use in febrile neutropenia before and after intervention.

Baseline characteristics of the audited cases in 2024 are shown (Table 1). A total of 164 distinct episodes of febrile neutropenia were audited in 2024. There were 62 cases pre-intervention, and the remaining 102 cases were post-intervention from July to December 2024. Following implementation of the de-escalation protocol and accompanying intervention, the median rate of inappropriate antibiotic use decreased to 16.7% – 63.63% (median 23.35%, interquartile range 19% – 39.5%) from July–December 2024 (Figure 2). The reasons for inappropriate antibiotic use did not differ significantly pre- or post-intervention, but there appeared to be a smaller proportion of patients where antibiotics having been continued despite a clear alternate diagnosis (6/33 [18.2%] vs 3/29 [10.3%]), but an increase in the proportion of patients who received a prolonged duration of antibiotics (2/33 [6.1%] vs 6/29 [20.7%]). Continuing antibiotics for on-going neutropenia despite clinical improvement and resolution of fever at 96 hours appeared to remain the commonest cause of inappropriate antibiotic use (22/33 [66.7%] vs 18/29 [58.6%]). Comparing the adverse events pre- and post-intervention, there were no significant increase in in-hospital mortality (pre- versus post-intervention, 7/62 [11.3%] versus 5/102 [4.9%], p=0.214), need for intensive care (7/62 [11.3%] versus 3/102 [2.9%], p=0.030) or *Clostridioides difficile* infections (2/62 [3.2%] versus 4/102 [3.9%], *P* = 0.999) was observed, despite a decrease in inappropriate broad-spectrum antibiotic use.

**Table 1.** Summary of audited cases of febrile neutropenia from January to December 2024

Parameter	Pre-intervention (Jan-Jun, n = 62)	Post-intervention (Jul-Dec, n = 102)	*P*-value
Baseline characteristics			
Age (years)	54.3 (±15.8)	60.4 (±15.6)	0.017
Sex (male)	39 (62.9%)	57 (55.9%)	0.376
Hematology condition			0.325
Stem cell transplantation	8 (12.9%)	19 (18.6%)	
Hematologic malignancy (without transplant)	54 (87.1%)	82 (79.4%)	
Duration of neutropenia (days)	10.8 (±5.4)	11.0 (±7.9)	0.825
Presence of bacteremia	13 (21.0%)	29 (28.4%)	0.288
*Outcomes*			
Inappropriate antibiotic use	33 (53.2%)	29 (28.4%)	0.001
Reasons for inappropriate antibiotic use			0.334
Alternate diagnosis identified (non-bacterial infection, e.g. viral infection)	6/33 (18.2%)	3/29 (10.3%)	
Clinical improvement, resolution of fever at 96 hours and negative cultures	22/33 (66.7%)	17/29 (58.6%)	
Antibiotic spectrum too broad, not tailored according to culture results	3/33 (9.1%)	3/29 (10.3%)	
Antibiotic duration too prolonged, out of proportion to clinical syndrome	2/33 (6.1%)	6/29 (20.7%)	
In-hospital mortality	7 (11.3%)	5 (4.9%)	0.214
Need for intensive care	7 (11.3%)	3 (2.9%)	0.030
*Clostridiodes difficiles* infection	2 (3.2%)	4 (3.9%)	0.999

Qualitatively, we found that the monthly feedback and report cards were well-received, fostering a culture of accountability and continuous improvement. The use of the protocol was perceived to improve confidence in de-escalating antibiotics when it was appropriate. It was also found to be simple to apply in clinical settings.

## Discussion

This quality improvement project highlights the potential for a structured intervention to have a transformative impact in addressing inappropriate antibiotic use amongst patients with febrile neutropenia. The introduction of a clear and evidence-based de-escalation protocol that was developed in conjunction with hematologists successfully helped to bridge knowledge gaps and provided clinicians with actionable criteria to guide decision-making.^2,6^ The protocol was easily accessible on the hospital intranet, which physicians referred to for the management of febrile neutropenia. The easy accessibility of this protocol helped ensure consistent application of stewardship principles while minimizing variability in practice.

In the design of the protocol, the key stakeholders, including hematologists were involved in the process. The commonest possible clinical scenarios were included clearly in the protocol (Figure 2) to ensure that it was easy to apply in a clinical setting. In situations that were gray, for example, in the setting of persistent fever despite broad-spectrum antibiotic therapy, infectious diseases consultation was recommended, for the specialists to help guide antimicrobial therapy and further investigations.^9^ This collaborative approach helped to foster confidence in the safety of the protocol, leading to improved uptake.

Importantly, the protocol needed to be supported by educational interventions and efforts to effectively engage stakeholders and addressing entrenched attitudes toward antibiotic use. Workshops and case discussions provided opportunities for clinicians to voice concerns and gain confidence in de-escalation strategies, ultimately fostering a culture of stewardship. This is a common feature as well in antimicrobial stewardship interventions in other similar settings.^10,11^ However, in addition to these initial education efforts, our project also implemented a continual feedback system. The integration of these feedback mechanisms, which includes a monthly audit and “report cards” provided to the hematology, further enhanced the project’s impact by promoting accountability and facilitating data-driven improvements. These regular audits not only tracked progress but also reinforced adherence to the antibiotic de-escalation protocol. Each feedback session highlighted illustrative cases where antibiotics were appropriately de-escalated, and also included cases where there had been room for improvement. Our findings align with existing literature demonstrating that feedback loops are essential in sustaining behavior change within clinical teams.^12,13^ Importantly, the protocol also encouraged the involvement of infectious diseases physicians in the management of complex cases of persistent fever, in order to provide guidance on appropriate investigations and antimicrobial therapy. It is also possible that reduction in the indiscriminate use of broad-spectrum antimicrobials would help to reduce the selection pressure on MDROs, and reduce colonization rates and invasive infections with these MDROs amongst hematology patients in the longer term.^14^

The protocol could be improved further. It appeared that the protocol was most effective in guiding physicians to stop antibiotics when an alternate diagnosis was made (e.g. if the patient had been diagnosed with a confirmed viral illness, or drug fever). This proportion of patients on inappropriate antibiotics for this reason dropped by nearly half following the intervention. However, further work with the hematology teams would be focused on encouraging cessation of antibiotics when there is clinical improvement and resolution of fever at 96 hours, despite on-going neutropenia, which was the reason that continued to account for more than half of the inappropriate antibiotic use post-intervention.^15^ Antibiotic discontinuation in this context is supported by prior studies and international guidelines.^15–17^ The proportion of patients who received in inappropriately long duration of antibiotics for their clinical syndrome had also increased from 6.1% to 20.7%. Guidance on appropriate antibiotic duration for specific clinical syndromes are available on the hospital intranet in a separate document, but could be incorporated in a streamlined fashion into our protocol for ease of reference and improved adherence.^18^

We had also observed some instances where the patient experienced clinical deterioration following initial adherence to the protocol with appropriate cessation or de-escalation of antibiotics. In such complex cases, we encouraged close collaboration between the hematology and infectious diseases teams. These episodes of deterioration were often multifactorial, encompassing a new or relapsed infection, progression of the underlying cancer or illness or other treatment-related toxicities. The simple protocol that we had implemented was a useful guide for antibiotic decision-making, but could not be blindly applied to each patient. For complex cases of febrile neutropenia, it was the close collaboration between hematology and infectious diseases in the management of patients that helped with optimal antimicrobial choices and investigations.

Additionally, several other limitations remain to our study. The relatively small sample size from our single-center experience may have limited our ability to observe statistically significant differences in outcomes. Furthermore, this was designed as a ‘before and after’ study, which increased the likelihood that study participants following implementation may receive a higher level of scrutiny than those before, which introduces a degree of bias.

In terms of implementation and uptake, an important barrier we had observed was the initial hesitation among junior staff to de-escalate antibiotics due to fear of adverse outcomes. Particularly, in after-hours settings, there was also a tendency to initiate or escalate antibiotics in the presence of persistent fever. This highlights the need for ongoing mentorship to bolster clinician confidence, in order to make decisions on antibiotic choices based on clinical assessment. Additionally, systemic issues such as frequent turnover of the junior staff would also mandate the need for frequent educational sessions to improve knowledge of this protocol. Addressing these factors through streamlined processes, as well as digital integration of this protocol could further enhance the feasibility and uptake of antibiotic de-escalation when clinically indicated. More specifically, incorporating this protocol into an electronic order set within the medical records could help to ensure adherence to this protocol.^19^

Overall, although the proportion of patients receiving inappropriate antibiotics has fallen significantly, approximately a quarter of patients still receive inappropriate antibiotics. Therefore, further work is needed, with close collaboration between hematology and infectious diseases to continue to refine our processes. For such a study and intervention focusing on behavioral change, it was also critical to ensure long-term sustainability of this intervention. Continued efforts are required to continue to monitor and measure the long-term impact of these interventions as well. The sustainability of these improvements will depend on maintaining momentum through periodic education, robust auditing systems, and continued stakeholder engagement. There may also be long-term implications in altering the prevalence of MDRO colonization amongst hematology patients. Furthermore, following the successful implementation of this amongst hematology patients, future work may include expanding the intervention to other disciplines managing immunocompromised patients, such as oncology and rheumatology.

In summary, we found that this project demonstrates that a well-structured and collaborative approach can significantly reduce inappropriate antibiotic use without compromising patient safety. By addressing both behavioral and systemic factors, the intervention was able to optimize antimicrobial stewardship practices and also gives rise to the potential for broader applications across other healthcare settings.

## Conclusions

In our single, tertiary center experience, the implementation of a simple-to-use antibiotic de-escalation protocol, supported by education and feedback mechanisms, significantly reduced inappropriate antibiotic use in febrile neutropenia among hematology patients, without compromising patient safety.

## Data Availability

Data may be made available on request from the corresponding author.
